# The legacy of industrial pollution in estuarine sediments: spatial and temporal variability implications for ecosystem stress

**DOI:** 10.1007/s10653-019-00316-4

**Published:** 2019-05-22

**Authors:** Kiri Rodgers, Iain McLellan, Tatyana Peshkur, Roderick Williams, Rebecca Tonner, Charles W. Knapp, Fiona L. Henriquez, Andrew S. Hursthouse

**Affiliations:** 1 School of Health and Life Sciences, University of the West of Scotland, Paisley, PA1 2BE UK; 2 School of Computing, Engineering and Physical Sciences, University of the West of Scotland, Paisley, PA1 2BE UK; 3 Department of Civil and Environmental Engineering, Centre for Water, Environmental, Sustainability and Public Health, University of Strathclyde, Glasgow, G1 1XQ UK

**Keywords:** Legacy pollution, Sediments, Metals, Ecosystems, Antimicrobial resistance

## Abstract

The direct impacts of anthropogenic pollution are widely known public and environmental health concerns, and details on the indirect impact of these are starting to emerge, for example affecting the environmental microbiome. Anthropogenic activities throughout history with associated pollution burdens are notable contributors. Focusing on the historically heavily industrialised River Clyde, Scotland, we investigate spatial and temporal contributions to stressful/hostile environments using a geochemical framework, e.g. pH, EC, total organic carbon and potentially toxic elements: As, Co, Cr, Cu, Ni, Pb and Zn and enrichment indicators. With regular breaches of the sediment quality standards in the estuarine system we focused on PTE correlations instead. Multivariate statistical analysis (principle component analysis) identifies two dominant components, PC1: As, Cr, Cu, Pb and Zn, as well as PC2: Ni, Co and total organic carbon. Our assessment confirms hot spots in the Clyde Estuary indicative of localised inputs. In addition, there are sites with high variability indicative of excessive mixing. We demonstrate that industrialised areas are dynamic environmental sites dependant on historical anthropogenic activity with short-scale variation. This work supports the development of ‘contamination’ mapping to enable an assessment of the impact of historical anthropogenic pollution, identifying specific ‘stressors’ that can impact the microbiome, neglecting in estuarine recovery dynamics and potentially supporting the emergence of antimicrobial resistance in the environment.

## Introduction

Industrial activities, whether contemporary or historical, have often occurred globally along major watercourses, particularly in estuaries where transport hubs and habitation sites have developed over civilisation. These features are also naturally chemically and physically important at the interface between terrestrial and marine systems where high flux of natural geochemical transport occurs, stimulating biodiversity and supporting critical ecosystems. The consequence has often impaired water quality, habitat loss and diminished ecosystem services, which result in deleterious changes in ecosystem structure with risks to human and aquaculture health. These changes could result in toxicologically stressful environments that, in turn, could be related to changes in microbiome and/or resistance—e.g. development of antimicrobial resistance (AMR) (Rodgers et al. 2018). Recovery of estuaries from long-term anthropogenic disruption is understood in some detail for higher trophic levels and within that context shows great variation from months to decades depending on biological species or system components (Borja et al. 2010). Very little understanding exists on the microbiome response to pollution and its impact on other characteristics.

The Clyde Estuary, Scotland, is an optimal example site to determine environmental ‘stress’, historically being one of the most contaminated estuarine environments in the UK (Balls et al. 1997). Through its historical development in Industrial Revolution, trade and wider urban development has seen massive anthropogenic disruption over relatively short spatial scales. Despite the recent improvement in water column quality and positive response of higher trophic levels (Whitfield and Elliot 2002), sediment contamination is retained and slower to recover (Borja et al. 2010). Consequently, an extensive list of historical pollutants has been thoroughly studied (Hursthouse et al. 2003; Edgar et al. 2003; Hursthouse 2001; Fernandes et al. 2009; Lass-Evans et al. 2012; Rowan 2002). The River Clyde and its tributaries have been important vectors and sink for contaminated waste from succession of industries located in the conurbation of Glasgow and along the banks of the estuary during the industrial development phase in Western Europe in the nineteenth and twentieth centuries. Consequently, the river has received pollution from the onset of the Industrial Revolution (eighteenth century) to the present day, changing with urban and industrial development in its catchment (Vane et al. 2007, 2010; Edgar et al. 2006). Metals such as As, Co, Cr, Cu, Ni, Pb and Zn (and other potentially toxic elements—PTEs) can be linked to anthropogenic and natural inputs (Caccia et al. 2003; Rodriguez-Iruretagoiena et al. 2016; Birch et al. 2015; Lenart-Boroń and Boroń 2014) which are now managed through a range of regulatory instruments, e.g. Water Framework Directive (2000/60/EC)(Cuculić et al. 2009; Khan et al. 2017; Larrose et al. 2010). The bioavailability and environmental influence of these pollutants depend on many processes such as (i) mobilisation in interstitial water, (ii) chemical species, (iii) transformation, e.g. methylation, (iv) sediment composition, e.g. oxides of Fe and organic matter, (v) competition with other metals for complexation, (vi) spatial distribution and (vii) influence of bioturbation, salinity, redox or pH on these processes (Bryan and Langston 1992; Weil and Brady 2016; Peng et al. 2008; Konhausera et al. 2002; Akcil et al. 2015; Zaaboub et al. 2015; Caccia et al. 2003; Berner and Berner 2012; Petit et al. 2015).

Being able to identify key correlations between physicochemical and PTE parameters is crucial in order to identify ‘stressors’ within the local environment. With past discharges to the River Clyde being reworked by estuarine processes and exposure conditions having a dynamic temporal context, there is a unique ecosystem to test the prevalence of pollution-related impacts. For example, the link between AMR and metal content which has previously been identified as a promoter of genetic dissemination by cross and/or co-resistance (Ashbolt et al. 2013; Baker-Austin et al. 2006; Berg et al. 2010; Seiler and Berendonk 2012; Perry and Wright 2013; Martinez et al. 2009; Knapp et al. 2017).

In this study, we explore metal levels and other geochemical characteristics along the River Clyde, Glasgow (Scotland), with an aim to support our baseline hypothesis that specific ‘stressors’ within the environment contribute risks to public health. Through identification of pollution conditions targeting locations with demonstrable exceedances of toxicity screening levels, we report the statistical significance of physicochemical parameters (pH, Ec, OM % and total organic carbon (TOC)) and key PTEs: As, Co, Cr, Cu, Ni, Pb and Zn within an industrialised estuary to identify key relationships (components). This research acts as a precursor to a larger study which will incorporate and correlate these results with microbial data.

## Analyses

### Sample collection

**Fig. 1:**
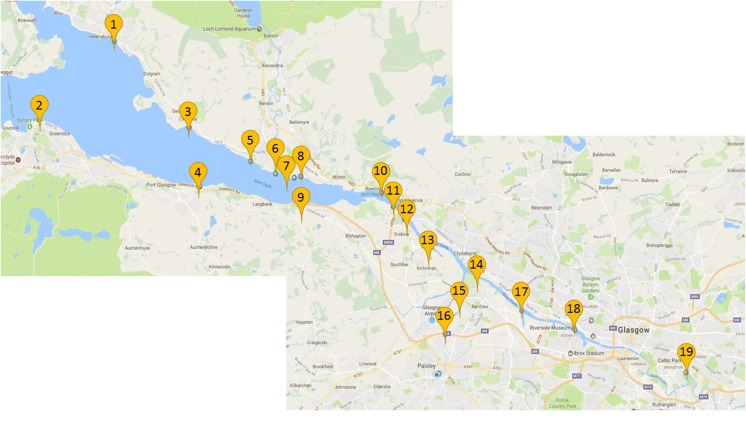
Location of sample sites along the River Clyde. Code + seaward distance from Glasgow tidal weir (km: 1: HNB: Helensburgh (33.17); 2: GNK: Greenock (37.9); 3: CAR: Cardross (28.47); 4: KBP: Kelburn Park (27.88); 5: DBW: Dumbarton West (25); 6: CYV: Clydeview (23.5); 7: DBC: Dumbarton Castle (22.45); 8: GRB: Gruggies Burn 22.05); 9: PGS: Port Glasgow (22.05); 10: BOW: Bowling (17.42); 11: BSP: Bishopton (16.44); 12: ESK: Erskine Harbour (15.5); 13: NSI: Newshot Island (13.67); 14: RSD: Rothesay Dock; 15: WCB: White Cart Bridge (4.75); 16: WCE: White Cart Estuary;17:  KGD: King George V Dock; 18: KLC: Kelvin Confluence; 19: CUN: Cuningar Loop (− 5.4)

Sediment samples were collected from 19 sites along the inner Clyde Estuary (Fig. 1) during sampling campaigns in Autumn 2017 to Spring 2018, to a maximum depth of 30 cm (handheld coring device; AMS, Inc.). Three cores were collected at each site: one from the high tide line, one from the low tidal front and one-half way between the two; each core was approximately 5 m apart. Four exceptions: Rothesay Dock, King George V Dock, White Cart Estuary and the Kelvin Confluence, were grab samples collected from the centre of the river. Samples were returned to the laboratory, and each core was subdivided into 10 cm sections, before homogenisation, with subsamples taken for microbiological analysis. The remaining sample was air-dried until constant weight and then sieved < 500 μm for normalisation. This provided nine subsamples per location.

### Physical and chemical properties

Organic matter (OM) was determined using loss on ignition with total organic carbon (TOC) using van Bemmelen factor (Dean 1974; Salehi et al. 2011); pH and electrical conductivity were measured using calibrated Mettler Toledo FiveGo meters (Corwin and Rhoades 1982; Van Reeuwijk 2002). Moisture content was determined using a Mettler Toledo HB43-5 m. All analysis was carried out in replicate (*n* = 5).

### Elemental analysis

Replicate aliquots (*n* = 5) of 0.25 g ± 0.05 g of sample were treated with 5 ml 30% H_2_O_2_ overnight to remove organic matter in digestion cups (Environmental Express, USA) and then digested using *aqua regia* at 95 °C in a hot block until 5 ml in volume. They were then made up to 50 ml using UHP water and filtered in situ using 0.45-μm PTFE filter (FilterMate, Environmental Express, USA). A Certified Reference Material (CRM) was included in the digestion method (BCR667 Estuarine Sediment BCR^®^; Sigma Aldrich).

Elements were determined by inductively coupled plasma mass spectrometry (ICP-MS) (Thermo X-Series II). A calibration series of multi-element standard (BCR320R-40G—channel sediment) was determined regularly (every 60 samples + linearity *r*^2^ > 0.99) with indium used as the internal standard. Each determination was made in quadruplicate, and dilutions were made if necessary. Isotopes used for measurement were ^75^As, ^59^Co, ^52^Cr, ^63/65^Cu, ^60^Ni, ^206/207/208^Pb and ^66/67^Zn. Results were subjected to the geoaccumulation index in order to gauge the degree of anthropogenic influence.

## Results

Limits of detection were (mg/kg, dry weight): As: 0.10, Co: 0.012, Cr: 0.024, Cu: 0.082, Ni: 0.056, Pb: 0.01 and Zn 0.45 mg/kg with recoveries between 85 and 110%. Physical properties and PTE concentrations are given in Table 1, with comparable data for sediment quality criteria shown in Table 2. Statistical analysis was carried out using SPSS, with data tested for normal distribution using Kolmogorov–Smirnov and Shapiro–Wilk tests; PTE concentrations were log-transformed for analysis of variance (ANOVA) and principal component analysis (PCA).

**Table 1:** Sediment characteristics averaged across all sample cores, depths and locations

	As	Co	Cr	Cu	Ni	Pb	Zn
TEL	5.9		37.3	36.8	18	35	123
PEL	**17**		**90**	**197**	**35.9**	**91.3**	**315**
Helensburgh	5.1 (0.6)	4.5 (1.5)	21.5 (6.5)	13.9 (4.5)	17.1 (6.4)	26.0 (6.5)	66.4 (12.6)
Greenock	3.9 (0.8)	2.9 (0.5)	**164.6 (0.92)**	**199.4 (24.0)**	*32.1 (4.2)*	**103.8** (18.4)	*125.1 (15.7)*
Cardross	2.2 (0.5)	2.3 (0.05)	15.6 (1.6)	6.3 (0.5)	8.7 (0.6)	10.8 (1.4)	40.2 (3.0)
Kelburn Park	*7.8 (0.9)*	8.6 (0.9)	*38.7 (4.0)*	22.4 (1.9)	23.7 (2.1)	26.4 (6.1)	80.9 (12.3)
Dumbarton West	4.7 (1.6)	5.8 (0.6)	32.1 (2.55)	11.4 (0.7)	25.3 (1.7)	28.1 (11.8)	42.2 (9.8)
Clydeview	5.6 (0.7)	8.5 (1.2)	*47.3 (5.0)*	16.3 (1.5)	27.2 (3.4)	21.5 (4.3)	60.3 (3.7)
Dumbarton Castle	*13.3 (2.8)*	7.2 (0.6)	**92.0** (24.0)	25.0 (4.0)	23.0 (1.5)	*59.0 (15.7)*	105.3 (19.5)
Port Glasgow	6.8 (0.6)	10.2 (0.3)	36.4 (1.4)	16.7 (0.7)	*32.3 (0.9)*	11.4 (1.8)	54.0 (4.3)
Gruggies Burn	*10.9 (5.2)*	5.5 (0.8)	*40.7 (7.3)*	18.9 (3.1)	17.6 (1.1)	*36.1 (9.5)*	82.4 (7.2)
Bowling	2.0 (0.8)	7.6 (0.2)	16.5 (1.2)	9.9 (2.1)	18.1 (0.8)	19.88 (3.1)	45.6 (12.9)
Bishopton	4.1 (0.6)	4.6 (0.2)	*38.0 (1.5)*	10.8 (0.9)	12.5 (0.7)	23.3 (1.8)	51.5 (4.6)
Erskine	3.5 (1.0)	5.2 (0.4)	*68.1 (6.4)*	19.9 (2.1)	17.8 (1.9)	32.8 (5.0)	(12.0)
Newshot Island	**19.5** (3.1)	5.9 (0.6)	*44.4 (5.9)*	*136.6 (29.4)*	22.4 (2.1)	**106.6** (20.9)	*212.5 (37.3)*
Rothesay Dock	**18.8** (4.2)	12.0 (2.3)	**158.8** (20.1)	73.7 (4.1)	**39.5** (3.2)	**122.9** (13.8)	(37.0)
White Cart Bridge	4.7 (0.6)	17.0 (1.7)	*52.4 (1.3)*	23.3 (1.6)	**46.5** (1.9)	*38.2 (3.9)*	77.7 (4.4)
White Cart Estuary	7.2	4.5	25.4	11.5	14.4	16.2	58.7
King George V Dock	*15.7 (0.3)*	13.8 (2.2)	**153.6** (31.9)	*86.6 (7.4)*	**44.9 (1.4)**	*91.0 (10.4)*	**343.4** (5.4)
Kelvin Confluence	10.2	11.6 (1.9)	*57.4 (8.8)*	33.1 (2.5)	**39.1** (3.2)	*60.4 (7.7)*	*183.0 (14.4)*
Cuningar Loop	3.2 (0.5)	6.7 (0.2)	21.1 (1.0)	13.3 (1.5)	*20.5 (0.3)*	30.6 (4.0)	75.0 (6.0)

Concentrations above the threshold effect level (TEL) are in shaded cells, and above the probable effect level (PEL) is identified in bold. Values are dry weight mg/kg (95% confidence interval)

**Table 2:** IUGS/IAGC Global Geochemical Baselines compared to sediment quality guidelines (metal concentrations mg/kg)

	G-Baseline^a^ (Salminen et al. 2005)	Clyde (Jones et al. 2018)	Median	Min.	Max.	The Clyde (Krom et al. 2009)	TEL	PEL
pH	5.5–7.5		6.9	4.2	7.7			
TOC (%)	1.71		3.21	0.44	12.45			
Conductivity (mS)			1.41	0.41	19.95			
As	6–10.1	19	4.7	0.00	32.53		5.9	17
Co	8.0		7.0	2.48	30.10			
Cr	21–63	94	50	0.0	185.44		37	90
Cu	14–17	16	24	3.34	314.73	90–137	37	197
Ni	16–21	43	21	8.74	58.09		18	36
Pb	14–21	19	44	4.11	222.71	109–183	35	91
Zn	60–71	83	105	10.27	448.88		123	315

*TEL* threshold effect level, *PEL* probable effect level

^a^G-Baseline refers to the median concentrations (mg/kg) measured for European geochemical stream sediment baseline maps (Ccme 2001)

### Geoaccumulation index (*I*_geo_)

To provide further indication of combined contamination status, the *I*_geo_ was calculated using the methods of Muller (1979) and Abrahim and Parker (2008):

$$I_{\text{geo}} = \log_{2} \left( {\frac{{C_{n} }}{{1.5 B_{n} }}} \right)$$

where *C*_*n*_ is the concentration of the metal of interest in sediment sample, *B*_*n*_ is the background level of the same metal, and 1.5 is the factor to minimise the effect of possible variations in the background (Al-Haidarey et al. 2010; Okedeyi et al. 2014; Hasan et al. 2013).

**Fig. 2:**
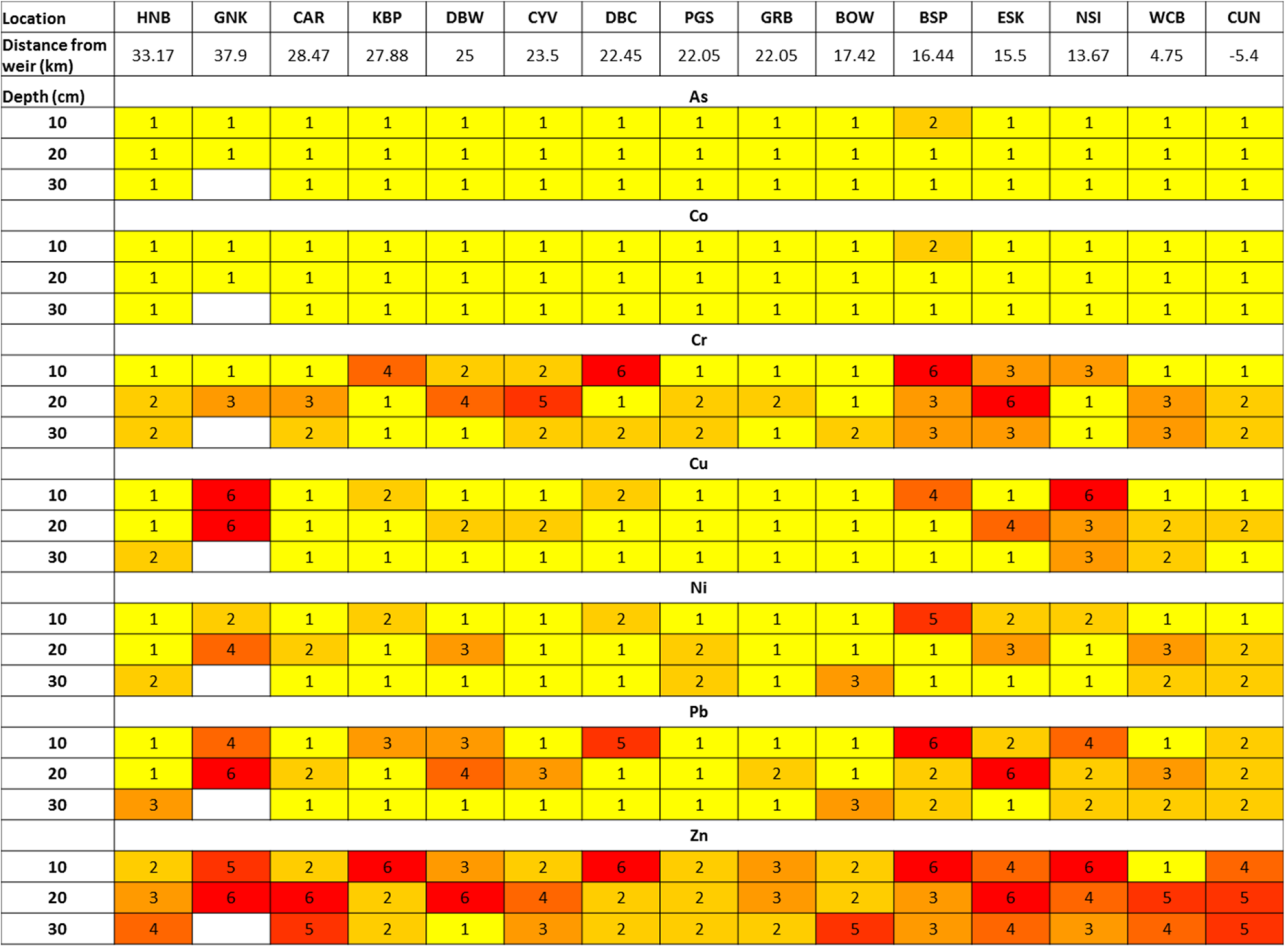
Heat map showing summary of geoaccumulation data along the River Clyde (downstream from the tidal weir in central Glasgow) and various depths (0–10 cm, 10–20 cm and 20–30 cm)

And evaluated against seven degrees of metal pollution in terms of seven contamination classes (see Fig. 2), with < 0 demonstrating no contamination and > 5 extremely contaminated.

IGO data show that all sediment samples (including at varying depths) are contaminated with low to extreme levels of contamination for all PTEs. However, this varied widely between PTE and distance/depth.

As and Co show low levels of contamination along the hole of the Clyde historically, i.e. with depth. Chromium predominantly shows an increase in contamination throughout history, whereas PTEs Cu, Ni, Pb and Zn show no discernible trend. Industrial trends throughout history, however, cannot be discounted without radiometric dating. Zn is seen to be the most extremely contaminated with only moderate levels seen in Port Glasgow. This site shows the lowest level contamination trend for all PTEs. There is varying evidence, which suggests both an accumulation in PTEs over time and an insight where contamination may have occurred. We can see that the high levels of contamination are seen between 25 and 33 km from the weir and again 16.44 km to the weir. These include GNK, CAR, KBP and DBW at the estuaries widest and again BSP, ESK, NSI, WCB and CUN, demonstrating zonal relationships (Fig. 1).

Analysis of variance (ANOVA) with Kruskal–Wallis testing was used to determine whether there were any significant differences in PTE concentration with sample location, depth and distance from the high tide mark (core number) (Table 3). There are significant differences (*p* < 0.001) between the locations for all PTEs, but there is no difference in the distance from the high tide line, i.e. tidal influence does not significantly affect PTE concentration. With regard to depth, only Pb showed significant difference with sample depth.

**Table 3:** ANOVA *F* values for PTEs against sample location, depth and core

	As	Co	Cr	Cu	Ni	Pb	Zn
Location	7.58*	13.02*	14.33*	25.16*	10.93*	9.69*	11.47*
Depth	0.55	2.22	0.70	0.35	2.12	3.31*	1.33
Core	0.29	0.21	1.70	0.19	0.05	0.44	0.46

*p < 0.001

Multiple correlation analysis (Table 4) shows a significant positive correlation for most variables with the exception of core number, suggesting that the distance to the high tide level does not influence the PTE concentrations. Note the negative correlation between location and pH (highlighting the changing salinity of estuarine sediments) and depth with Pb and Zn.

**Table 4:** Physicochemical and PTE correlation matrix

	Location	Core	Depth	Moisture	pH	EH	OM	As	Co	Cr	Cu	Ni	Pb	Zn	TOC
Location	1.000														
Core	− 0.047	1.000													
Depth	− 0.123	0.087	1.000												
Moisture	0.575**	− 0.157	− 0.153	1.000											
pH	− 0.624**	0.045	0.333**	− 0.378**	1.000										
EH	− 0.240**	− 0.094	− 0.197*	0.500**	0.018	1.000									
OM	0.439**	− 0.130	0.004	0.834**	− 0.174*	0.382**	1.000								
As	0.104	0.018	0.065	0.287**	0.171*	0.123	0.322**	1.000							
Co	0.423**	0.035	0.359**	0.398**	0.029	− 0.019	0.549**	0.266**	1.000						
Cr	0.306**	0.082	0.006	0.557**	− 0.232**	0.219**	0.481**	0.469**	0.463**	1.000					
Cu	0.144	− 0.050	0.021	0.233**	− 0.145	0.119	0.376**	0.436**	0.178*	0.357**	1.000				
Ni	0.250**	0.024	0.361**	0.322**	0.086	0.021	0.518**	0.212*	0.797**	0.385**	0.479**	1.000			
Pb	0.269**	− 0.067	− 0.404**	0.336**	− 0.417**	0.185*	0.320**	0.389**	− 0.061	0.424**	0.626**	0.080	1.000		
Zn	0.318**	− 0.014	− 0.193*	0.484**	− 0.314**	0.253**	0.465**	0.547**	0.178*	0.559**	0.708**	0.298**	0.753**	1.000	
TOC	0.435**	− 0.136	0.000	0.839**	− 0.170*	0.398**	0.994**	0.317**	0.546**	0.483**	0.369**	0.515**	0.321**	0.466**	1.000

**Correlation is significant at the 0.01 level (2-tailed). *Correlation is significant at the 0.05 level (two-tailed). *n* = 150 for all except Eh *n* = 141; As *n* = 144

PCA was carried out using the correlation matrix and varimax rotation to elucidate the principal components (Alexander et al. 2008). Four principal components (PCs) with an eigenvalue > 1 were returned (Table 5) and moisture content was not included due the nature of the matrix, i.e. at or near saturation. The dominant factors (41% of total variance) were anthropogenic PTEs: As, Cr, Cu, Pb and Zn which are typical ‘urban’ elements (Fordyce et al. 2012) which were all positively related to each other. The remaining three PCs account for 41.9% of the total variance: PC2 shows relationships between Co, Ni and total organic content (19.6%), PC3 is pH (11.8%), and PC4 is conductivity (10.5%). Interestingly, Ni exceeds the G-base, threshold effect limit and probable effect limits (Table 2); this potentially highlights the importance of their roles as essential nutrients.

**Table 5:** Principal component analysis with varimax rotation of all samples

	PC1	PC2	PC3	PC4
pH	− 0.206	0.031	**0.926**	0.034
EC	0.112	− 0.037	0.06	**0.962**
As	**0.769**	0.084	0.478	0.024
Co	0.108	**0.943**	0.061	− 0.061
Cr	**0.697**	0.339	− 0.14	0.088
Cu	**0.785**	0.371	− 0.013	0.019
Ni	0.133	**0.951**	0.091	− 0.043
Pb	**0.807**	− 0.116	− 0.36	0.129
Zn	**0.879**	0.09	− 0.182	0.141
TOC	0.285	**0.646**	− 0.206	0.463
Variability %	41.0	19.6	11.8	10.5

KMO: 0.703; Bartlett’s test of sphericity: > 0.05

## Discussion

PTE concentrations (Table 1) were compared to a number of previous studies, including the European Geochemical Baseline, Clyde waters and estuary sediments (Jones et al. 2018; Krom et al. 2009), the IUGS/IAGC Global Geochemical Baseline and the FOREGS (Forum of European Geological Survey) (Salminen et al. 2005). The Global Geochemical Baselines are the median levels of PTEs we expect to see in stream sediments. FOREGS produced a standardised method of sampling, chemical analysis and data management across 26 countries to produce national baseline datasets for comparable measures. These do not regulate or highlight parameter impact; rather, it allows a comparison with broad expected values for terrestrial surface materials (Table 2) (Salminen et al. 2005).

The comparison showed that values from this study exceeded the geochemical baseline values and were more comparable to the Clyde Estuary baseline study results, suggesting that levels exceeding the baseline are likely affected by anthropogenic inputs. As previously reported, estuarine sediments in industrialised regions create environmentally stressful conditions for the sediment microbiome (Rodgers et al. 2018); we therefore focussed on ‘stressors’ and concentrations above the threshold effect level (TEL) and probable effect level (PEL). The TEL represents the concentration below which adverse effects are unlikely to occur, whereas the PEL defines the level in which adverse effects are expected to occur (Macdonald et al. 2000).

It can be seen in Table 1 that As, Co and Cr median values are within the expected geochemical baseline, while Cu, Ni, Pb and Zn are above typical levels. The physicochemical parameters (pH and TOC) show that pH sits within typical baseline values, whereas organic carbon content varies greatly. Water content, conductivity and organic matter show a vast variety in concentrations and vary depending on the sampling site.

Newshot Island, Rothesay Dock, King George V Dock and the Kelvin Confluence exceed the TEL for all, if not most, of the PTEs. This could be an artefact of the different sampling techniques employed at the latter three; these were grab samples collected from the centre of the river rather than within the tidal area; or it could be due to the heavy industrialised presence at these locations. Newshot Island is on the opposite bank to one of Scotland’s largest wastewater treatment works which is a potential source of pollution in this area (Rodriguez-Iruretagoiena et al. 2016; Edgar et al. 2003). The PEL is exceeded at a number of locations: Greenock (Cr, Cu and Pb), Dumbarton Castle (Cr), Newshot Island (As, Pb), Rothesay Dock (As, Cr, Ni, Pb), King George V Dock (Cr, Ni, Zn) and Kelvin Confluence (Ni).

There is no spatial trend in As distribution; however, there are a number of hot spots, e.g. Newshot Island and Rothesay Dock where levels exceed the PEL. Historically, As was used to treat wood at timber and shipyards, both of which are known to have been near these locations (Clydewaterfront 2014). Elevated Cr levels at Dumbarton Castle (as well as a *I*_geo_ level of ‘extremely polluted’), Rothesay Dock and King George V Dock may also be linked to shipyards and timber yards as well as leather tanning (Muirhead 2014), particularly associated with known historic pollution levels in the Vale of Leven. Cu is predominantly below the TEL for most sample sites with little variation; the greatest variability is seen at Greenock and Newshot Island, with the former showing up to 13% variability, half of which exceed the PEL.

Elevated levels of Ni at the same site (average of 33.9 mg/kg) can be linked to Cu-Ni alloys typically used in ship building. With Ni there are also hot spots in areas of similar industrial heritage: Rothesay Dock, White Cart Bridge, King George V Dock and Kelvin Confluence. It is also worth noting that the Ni threshold effect level is exceeded at all but five locations, in conjunction with being moderately–extremely polluted (*I*_Geo_, Fig. 2) which will impact the microbiome. There is no clear trend with Co, but there is high variability in concentrations (2.3–15 mg/kg), with only the higher > 11.2 mg/kg to be considered above the natural baseline. The highest concentration of Zn was found at King George V Dock, and this was the only location to exceed the PEL. Zn, however, has the most polluted Igeo factor suggesting a strong human influence.

There appears to be no pattern in PTE concentration as the locations progress from saline conditions (Helensburgh) to freshwater conditions (Kelvin Confluence). Our control site at Cuningar Loop exceeded the TEL for Ni and three sample sites did not exceed the TEL for any PTE: Helensburgh, Cardross and the White Cart Estuary. These locations are all areas of low-current industrial activity compared to other sites. Changes in concentration are also reflected in the minimum and maximum values, with the maximum values exceeding regulatory and typical values. A comparison with a recently published study (Jones et al. 2018) which reported on summary data for samples collected in campaigns nearly two decades earlier from the Firth of Clyde (saline conditions) found that their concentrations are higher than our studies; however, our most comparable site (Greenock) exceeds the TEL for Ni and Zn and the PEL for Cr, Cu and Pb. Greenock also has the highest concentrations of Cr and Cu in our study (Table 1). A major difference between our study and Jones et al. (2018) is in relation to chromium; Jones et al. reported the majority of locations exceeded the PEL, whereas although twelve of our sites exceeded, the TEL only four exceeded the PEL.

As the Clyde Estuary is an ‘open system’, it is possible that there has been natural attenuation of historical pollution in the upper sediment column, particularly as Jones et al. (2018) collected samples from a greater depth. A greater column depth would not only reflect an earlier period in time, but also reflect more clay condition and possibly more ‘stable’ conditions, i.e. sediments that are not as affected by tidal changes. In our study the uppermost sediments were ‘jelly-like’ in their composition and are constantly being washed which could reduce higher PTE concentrations in areas where there are reduced industrial inputs. This therefore has implications for a potential increase in antimicrobial resistance due to exposure of historical pollution at depth (Rodgers et al. 2018). The time difference between our campaign and previous reports, particularly for Cr, shows the dynamic nature of the system with regard to complexity and elemental movement. It is widely reported that there are localised upstream sources of Cr contamination (Whalley et al. 1999); in the last decade, extensive engineering associated with transport infrastructure and international athletics events has transformed some of the most significant sources of Cr pollution with subsequent knock on effect downstream.

PCA analysis highlighted two prominent groupings relating to anthropogenic and natural sources; Component 2 (Co, Ni and TOC) we suggest is the natural and biological requirements within ecosystems because they are less commonly a consequence of anthropogenic causes. However, Co and Ni are typically distributed uniformly within sediments, their concentration increases with depth, and certain locations are likely to be as industrial depositions have accumulated over time (Leyssens et al. 2017; Cempel and Nikel 2006).

These results suggest that changes in salinity and proximity to high tide level have little impact on concentration; however, there are a number of pollution hot spots in the Clyde Estuary (Greenock, Newshot Island, Rothesay Dock, King George V Dock and the Kelvin Confluence) suggestive of site-specific inputs. In addition, there are sites with high variability which can be attributed to many factors including sediment mixing with historical inputs. Observations of the potential impact of upstream interventions, in particular on the input of chromium, highlight the dynamic nature of the system and still the system acts as a net pollution sink.

## Conclusion

Understanding the distribution of PTEs and geochemical factors in an industrialised estuary is important to elucidate the contributions to stressful/hostile environments. We see levels of PTEs often exceed TEL/PEL levels at various locations and depths along the Clyde, although they are starting to reduce in comparison with historical levels. Our PCA analysis identified four components with PC1 and PC2 accounting for the highest variance and associations: As, Cr, Cu, Pb and Zn as well as Ni and Co with TOC. Compared to extensive assessment of sediment quality in the region over more than a decade ago, our data show there has been a gradual reduction in sediment contamination levels, and for example impact of upstream source control (extensive remediation of Cr contamination in Glasgow). However, significant exceedances of sediment quality standards still occur in the estuary and are spatially variable.

It is evident that the estuarine sediments are still a reservoir of contaminants resulting in persistent exposure and disruption to the microbial consortia. Further research is required in order to determine whether these components can be identified as ‘stressors’ within an environmental microbiome. If identified correctly, does this subsequently exacerbate the prevalence of genetic mutations and more specifically antimicrobial resistance? The ‘contamination map’ created within this study provides the basis to enable a detailed study of the impact on the microbiome and causes the emergence of antimicrobial resistance in the environment.
